# A novel approach for detection of primary tooth caries based on the light influence foundation

**DOI:** 10.1186/1878-5085-5-S1-A123

**Published:** 2014-02-11

**Authors:** Anatoly A Kunin, Natalia S Moiseeva

**Affiliations:** 1 Professor, Dr. Med.Sc., Voronezh N.N. Burdenko State Medical Academy, Therapeutic Dentistry Department, Voronezh, Russia; 2 Voronezh N.N. Burdenko State Medical Academy, Therapeutic Dentistry Department, Voronezh, Russia

## 

Several techniques are used for detection of dental caries including a fiber-optic light (FOL), the method of optical coherence tomography (OCT), the method of fiber optic transillumination and the infrared tomography. For an assessment of light-induced fluorescence of hard dental tissues and primary dental caries diagnosis, we propose to use the domestic light-emitting-diode activator “LED active”, (MEDTORG +, Russia), with a wavelength of 530 nanometers and illumination of 10 000 meter-candle, and also wavelength of 625 nanometers at the 140 MWt/cm^2^ radiation power density. The activator’s principle action is based on application of light of powerful light-emitting diodes with the big intensity of a luminescence of monochrome color without a thermal component.

The present study employed 150 18-45 years old subjects. For the diagnosis and severity of initial caries we used – light-induced fluorescence and electrometric diagnostic tools. Caries prevention was attempted by daily applications of remineralized preparation – "Radogel-GAMK" with amino acids and hyaluronic acid for the caries precaution at the early stages for 15 days.

Under the influence of a "Radogel-GAMK"’s preparation parameters of electrometry at group of surveyed patients with initial caries have decreased by one third on hillocks, in fissures and on seal border. On fragment enamel and vestibular surfaces - in half, accordingly.

Under the influence of green light (530 nm) on the demineralized enamel area, zone of increased opacity was identified and, confirmed by the presence of microbial contamination and degradation products. The extent of opacity of demineralized enamel correlated with the process of demineralization and the index of fluorescence signal (+). The use of red light (625 nm) allows identifying secondary caries on borders of abutment filling, which is manifested as a center of brown pigmentation of varying intensity. Reducing opacity of demineralized tooth enamel under the green light impact, allowed assessment of the remineralization effect of dental hard tissues and restoration of its organic and mineral components.

As a result of research special parameters confirming the enamel changes after the impregnation by organic components that make up the protein matrix of the tooth have been identified. Remineralizing effect was proved by a reduction of electrometry parameters and opacity of demineralized tooth areas.

Thus, studies of light-induced fluorescence can offer an actual and effective method of early detection of initial carious lesions.

